# Minimum Acceptable Diet at 9 Months but Not Exclusive Breastfeeding at 3 Months or Timely Complementary Feeding Initiation Is Predictive of Infant Growth in Rural Bangladesh

**DOI:** 10.1371/journal.pone.0165128

**Published:** 2016-10-24

**Authors:** Aatekah Owais, Benjamin Schwartz, David G. Kleinbaum, Parminder S. Suchdev, A. S. G. Faruque, Sumon K. Das, Aryeh D. Stein

**Affiliations:** 1 Department of Epidemiology, Rollins School of Public Health and Laney Graduate School, Emory University, Atlanta, GA, United States of America; 2 Los Angeles County Department of Public Health, Los Angeles, CA, United States of America; 3 Department of Epidemiology, Emory University, Atlanta, GA, United States of America; 4 Hubert Department of Global Health, Emory University, Atlanta, GA, United States of America; 5 International Center for Diarrheal Disease Research, Dhaka, Bangladesh; Universidade de Sao Paulo, BRAZIL

## Abstract

The association between suboptimal infant feeding practices and growth faltering is well-established. However, most of this evidence comes from cross-sectional studies. To prospectively assess the association between suboptimal infant feeding practices and growth faltering, we interviewed pregnant women at 28–32 weeks’ gestation and followed-up their offspring at postnatal months 3, 9, 16 and 24 months in rural Bangladesh. Using maternal recall over the past 24 hours, exclusive breastfeeding (EBF) status at 3 months, age at complementary feeding (CF) initiation, and receipt of minimum acceptable diet (MAD; as defined by WHO) at 9 months were assessed. Infant length and weight measurements were used to produce length-for-age (LAZ) and weight-for-length (WLZ) z-scores at each follow-up. Generalized estimating equations were used to estimate associations of LAZ and WLZ with infant feeding practices. All models were adjusted for baseline SES, infant sex, maternal height, age, literacy and parity. Follow-up was completed by 2189, 2074, 1969 and 1885 mother-child dyads at 3, 9, 16 and 24 months, respectively. Stunting prevalence increased from 28% to 57% between infant age 3 and 24 months. EBF at 3 months and age at CF initiation were not associated with linear infant growth, but receipt of MAD at 9 months was. By age 24 months, infants receiving MAD had attained a higher LAZ compared to infants who did not receive MAD (adjusted β = 0.25, 95% CI: 0.13–0.37). Although prevalence of stunting was already high at age 3 months, ensuring infants receive a diverse, high quality diet from 6 months onwards may reduce rates of stunting in the second year of life.

## Introduction

According to the most recent global estimates, 165 million children less than 5 years of age are undernourished, with 52 million suffering from severe malnutrition [1]. Children with poor nutrition are also at increased risk of illnesses, such as diarrhea and respiratory infections [2], deficits in cognitive development, as well as diminished work capacity and increased risk of chronic diseases in adulthood [3]. Undernutrition is also the underlying cause of 3.1 million child deaths each year [1].

Infant and young child feeding (IYCF) practices are important determinants of nutritional status of children. The World Health Organization (WHO) recommends exclusive breastfeeding (defined as no other food or drink except for medicines and/or nutritional supplements) for the first six months of life, followed by introduction of complementary foods from age 6 months, with continued breastfeeding until the child is at least two years of age. Inadequate infant and young child feeding practices are increasingly recognized as major contributors to poor infant nutrition and growth faltering [4–6], and the prevalence of poor IYCF practices remains high in many parts of the world [7–9].

Bangladesh suffers from a disproportionately high burden of childhood undernutrition [10]. The prevalence of children less than 5 years of age with height-for-age, weight-for-age, and weight-for-height z-scores less than -2.0 is estimated to be 36%, 33%, and 14%, respectively [11]. This high burden of childhood undernutrition occurs concurrently with a high prevalence of inadequate IYCF practices. Only 55% of infants < 6 months are exclusively breastfed and only 7% of infants 6–8 months of age are fed according to WHO recommendations [11].

Given the high burden of childhood undernutrition in Bangladesh, there is a need for immediate remedial action, effective and sustainable at the national level. Inviting national governments to be major stakeholders, and including them in the planning and execution of IYCF interventions is essential for ensuring program scale-up, sustainability and success in achieving improvements in childhood growth measures [12, 13]. To contribute to the growing body of longitudinal evidence linking improvements in IYCF practices and subsequent childhood growth, both linear and ponderal, an integrated set of individual-, household- and community-level interventions was implemented in a rural sub-district of Bangladesh, in partnership with the national government. The objective of the present study is to evaluate the relationship between IYCF practices in the first year of life with subsequent childhood growth measures, in the context of rural Bangladesh.

## Subjects and Methods

### Study population and setting

As described in detail previously [14], data for this study were collected in the context of evaluation of Window of Opportunity, a community-based nutrition and infant and young child feeding program implemented by CARE in six countries, including Bangladesh, where it was known as *Akhoni Shomay*. *Akhoni Shomay* was carried out in Karimganj. A rural sub-district of Kishoreganj with a population of ~320,000, Karimganj is approximately 120 km north of Dhaka. Detailed descriptions of study setting, population and data collection tools have also been presented elsewhere [15, 16].

An integrated set of individual-, household- and community-level interventions was implemented. Women living in a second sub-district, Katiadi, served as controls. In the context of programmatic evaluation, a prospective cohort of ~2400 pregnant women (1200 per sub-district) was recruited between January and October 2011. Pregnant women were recruited in their 7th month of gestation, with follow-up of their offspring scheduled to occur at 3, 9, 16 and 24 months of age.

### Variable derivation

#### Infant diet

Exclusive breastfeeding (EBF) status at 3 months was assessed using maternal recall of what the child ate/drank in the past 24 hours. Infant age (in months) at complementary feeding (CF) initiation was estimated using maternal recall at 9-month follow-up. The indicator for assessing infant diet quality as developed by the WHO [17] was used to assess whether an infant received a minimum acceptable diet (MAD) at age 9 months. This variable is derived from the number of food groups as well as the number of meals consumed by an infant over a 24 hour period. The food groups include, grains, roots and tubers; legumes and nuts; dairy products; flesh foods; eggs; vitamin-A rich fruits and vegetables; and other fruits and vegetables. Furthermore, whether an infant received a minimum acceptable diet or not is also determined by his or her age and breastfed status. For example, a 9 months old breastfed infant should receive ≥ 4 food groups and ≥ 4 meals over 24 hours to be considered having received MAD. Using maternal recall at the 9-months’ follow-up, everything a child ate over the past 24 hours was categorized into one of seven food groups, as well as the number of meals consumed to determine whether s(he) received MAD.

#### Infant anthropometric measures

At each follow-up, trained study personnel measured each child’s length and weight using methods prescribed by WHO [18]. All anthropometric measurements were recorded in duplicate, with the average used in analyses. Age-adjusted length (LAZ) and weight Z-scores (WLZ) were calculated using the 2006 WHO Child Growth Standards [19].

#### Other covariates

Information on socio-demographic variables, including household socioeconomic status, maternal age, literacy and parity were abstracted from the questionnaire administered at recruitment into the study at 28 weeks’ pregnancy. Maternal height was measured to the nearest 0.1 cm by trained study personnel using stadiometers. Maternal height and infant sex was captured at the 3-month follow-up. Data on history of infant illness in two previous weeks was collected at each follow-up.

#### Statistical analysis

Data were imported into Statistical Analysis Software (SAS), version 9.3 for analysis. Median (range) was calculated for continuous variables and frequencies and percentages were calculated for categorical variables. Household socioeconomic status was assessed through residence characteristics and ownership of assets. Principal Component Analysis was used to create an asset based socioeconomic status score using methods described by Filmer and Pritchett [20].

To account for correlation between outcomes at different follow-up times from same child we used generalized estimating equation models. SAS procedure PROC GENMOD was used to generate β estimates and 95% confidence intervals (CI) for LAZ and WLZ, and odds ratios and corresponding 95% CI for stunting (LAZ < -2.00), using data from 9, 16 and 24 months of infant age. We assumed an autoregressive(1) (AR(1)) covariance matrix, with robust variance estimates. The AR(1) structure assumes a correlation between measurements from an individual that declines exponentially with time. To account for the evaluation design, indicator variables for district of residence and timing of enrollment into the cohort were also included in all models.

#### Ethics

This study was approved by the Research Review Committee (RRC) and the Ethical Review Committee (ERC) of the International Center for Diarrheal Disease Research, Bangladesh (icddr,b). At the time of enrollment, informed written consent was provided by each woman and a copy of the consent form was left with the participant. Verbal consent was obtained at each follow-up visit, with mothers/primary caregivers providing consent on behalf of the children included in the study. Obtaining written consent at these times was not deemed necessary as the study description provided at the start of the study included information about the follow-up visits. Consent procedures were approved by Research Review Committee (RRC) and Ethical Review Committee (ERC) of icddr,b.

## Results

A total of 2400 women were recruited at baseline, between January and October 2011. Follow-up was completed by 2189, 2074, 1969 and 1885 mother-child dyads at 3, 9, 16 and 24 months of infant age, respectively.

Table 1 summarizes the characteristics of study households stratified by district of residence. There were statistically significant differences in only two of the covariates of interest–at recruitment, a higher proportion of mothers in the intervention group could not read at all, and at the 3-month follow-up, a higher proportion of infants in the control district had a history of illness in the past 2 weeks.

**Table 1:** Selected characteristics of mother-child dyads in Kishoreganj, Bangladesh.

		Karimganj (Intervention)	Katiadi (Control)	
				*p*
**Maternal**^**a**^	N	1200	1200	
Age				0.05
≤ 24 years		50.3	55.3	
25–34 years		42.8	39.0	
≥ 35 years		6.9	5.7	
Height^b^, cm ± SD		150 ± 5.45	149.3 ± 5.24	0.19
Literacy				0.01
Cannot read at all		37.9	32.4	
Can read part of a sentence		13.9	16.8	
Can read a complete sentence		48.2	50.7	
Parity				0.46
1		27.4	28.1	
2		24.8	23.5	
≥ 3		47.9	48.4	
Socioeconomic status				0.59
1^st^ quintile (lowest)		19.5	20.5	
2^nd^ quintile		19.8	20.3	
3^rd^ quintile		21.0	19.0	
4^th^ quintile		20.6	19.4	
5^th^ quintile (highest)		19.2	20.8	
**Infant**^b^	*n*	1091	1102	
Female		50.5	50.1	0.85
History of illness in past 2 weeks		54.0	61.5	<0.001

Data in columns are percentages, unless otherwise stated

^a^Data are from recruitment at 28–32 week’s gestation

^b^Data are from the 3-month follow-up

The mean LAZ and WLZ scores at each follow-up are presented in Table 2. At 3 months 28% of infants were found to have LAZ < -2.00. This prevalence increased to 57% at 24 months.

**Table 2:** Nutritional status by infant age in Kishoreganj, Bangladesh.

Age (months)	3	9	16	24
**N**	2189	2074	1969	1885
**Mean LAZ (± SD)**	-1.50 (± 1.16)	-1.71 (± 1.16)	-2.08 (±1.11)	-2.21 (±1.06)
**Mean WLZ (± SD)**	-0.06 (± 1.20)	-0.53 (± 1.11)	-0.95 (± 1.02)	-0.82 (± 1.09)
**Stunting (%)**	28.1	35.9	51.5	56.9
**Wasting (%)**	4.6	7.6	13.2	10.1

Based on maternal report at 3 and 9 months follow-up, 45% of infants were exclusively breastfed at 3 months, timely CF was initiated for 49% of infants, whereas 44% of children in the study were introduced to complementary foods late. Even though 74% of infants received the minimum number of meals recommended by WHO during the past 24 hours, only 16% were given ≥ 4 (out of 7) of the WHO recommended groups. Consequently, only 16% of infants were receiving a minimum acceptable diet at 9 months of age.

The distribution of stunting and wasting by infant diet at 9 months is presented in Table 3. Wasting prevalence was similar among infants who did and did not receive MAD at 9 months, but prevalence of stunting was significantly lower at each follow-up among infants who received MAD at 9 months (p < 0.05). Prevalence of stunting and wasting did not differ by EBF status at 3 months or by age at which CF was initiated (data not shown).

**Table 3:** Infant feeding practices at age 9 months and prevalence of stunting and wasting in Kishoreganj, Bangladesh.

**Stunting**	**9 months**			**16 months**			**24 months**		
	*n*	% Stunted	*p*	*n*	% Stunted	*p*	*n*	% Stunted	*p*
Minimum meal frequency			<0.01			<0.01			0.01
≤ 3 meals	527	41.6		491	57.2		462	61.7	
≥ 4 meals	1542	33.9		1464	49.5		1386	55.1	
Minimum dietary diversity			0.02			<0.01			<0.01
≤ 3 food groups	1730	37.0		1630	52.8		1538	58.2	
≥ 4 food groups	339	30.1		325	44.9		310	49.7	
Minimally acceptable diet			0.02			0.01			<0.01
No	1736	37.0		1635	52.7		1544	58.2	
Yes	333	30.0		320	45.0		304	49.7	
	9 months			16 months			24 months		
**Wasting**	*n*	% Wasted	*p*	*n*	% Wasted	*p*	*n*	% Wasted	*p*
Minimum meal frequency			0.03			0.09			<0.01
≤ 3 meals	527	9.9		493	15.2		463	14.0	
≥ 4 meals	1543	6.9		1466	12.3		1388	8.8	
Minimum dietary diversity			0.08			0.18			0.05
≤ 3 food groups	1730	8.1		1633	13.5		1541	10.7	
≥ 4 food groups	340	5.3		326	10.7		310	7.1	
Minimally acceptable diet			0.09			0.22			0.07
No	1736	8.1		1638	13.4		1547	10.7	
Yes	334	5.4		321	10.9		304	7.2	

The results from GEE models are presented in Table 4. EBF status at 3 months or age at CF initiation were not predictive of linear or ponderal infant growth between ages 9–24 month, but receipt of MAD at 9 months was. Infants who received MAD at 9 months had a higher LAZ (adjusted β = 0.25, 95% CI: 0.13–0.37) and WLZ (adjusted β = 0.21, 95% CI: 0.10–0.33), in the second year of life, compared to infants who did not receive MAD at 9 months. Furthermore, female children were taller (adjusted β = 0.13, 95% CI: 0.05–0.21), compared to male children, and each additional cm of maternal height was associated with a 0.07 SD (95% CI: 0.06–0.08) gain in LAZ. For wasting, infants who had a history of illness in the two weeks preceding follow-up had a lower WLZ (adjusted β = -0.10, 95% CI: -0.15–-0.05), indicating that recent illness is associated with acute growth faltering.

**Table 4:** Association between infant feeding practices and infant nutritional status^a^ in Kishoreganj, Bangladesh–results from GEE models^b^.

	LAZ^c^	WLZ^c^^,^^d^	Stunting^c^
	β (95% CI)	β (95% CI)	OR (95% CI)
**Exclusive breastfeeding at 3 months**			
No	Ref.	Ref.	Ref.
Yes	0.08 (-0.002, 0.17)	0.06 (-0.03, 0.14)	0.91 (0.78, 1.07)
**Age at complementary feeding initiation**			
Early (≤ 4 months)	-0.03 (-0.17, 0.12)	-0.01 (-0.17, 0.14)	1.25 (0.92, 1.69)
Timely (5–6 months)	Ref.	Ref.	Ref.
Late (≥ 7 months)	-0.08 (-0.17, 0.004)	-0.04 (-0.12, 0.05)	1.23 (1.05, 1.44)
**Minimum acceptable diet at 9 months**			
No	Ref.	Ref.	Ref.
Yes	0.25 (0.13, 0.37)	0.21 (0.10, 0.33)	0.71 (0.58, 0.88)

^a^ LAZ: length-for-age z-score; WLZ: weight-for-length z-score

^b^ Include observations from 9, 16 and 24 months

^c^ Adjusted for SES, infant sex, maternal age, height, literacy, parity, district of residence and timing of enrollment

^d^ Also adjusted for history of infant illness in past 2 weeks

We also assessed whether recent morbidity was in the causal pathway between infant feeding practices and WLZ. Results from the reduced GEE models (excluding history of recent illness) were similar to those obtained from the full model. Therefore, we concluded that recent morbidity was not a mediator in the relationship between infant diet and acute growth faltering in our population.

We also assessed whether infants who received animal milk or other foods, in addition to breastmilk at 3 months, experienced poorer growth outcomes, compared to those who were exclusively or predominantly breastfed. There were no differences in LAZ, WLZ or odds of stunting among infants who were EBF or predominately breastfed compared to those who were partially breastfed or not breastfed at all.

## Discussion

This study used a prospective cohort to evaluate the association between IYCF practices in the first year of life and subsequent childhood growth outcomes. We found that only MAD at age 9 months, and not EBF at 3 months or timely CF initiation, was an independent predictor of linear and ponderal growth between 9–24 months of age.

Median duration of EBF in Bangladesh is < 4 months [21]. Therefore, it is not surprising that only 45% of infants in our cohort were being exclusively breastfed at 3 months. We also did not observe an association between EBF at 3 months and LAZ/stunting. This is not unforeseen. Although there is very strong evidence linking EBF with decreased risk of child morbidity and mortality [22, 23], the evidence for the same on child LAZ is less robust [24].

In our cohort, recent illness was a significant predictor of deficits in WLZ. This finding is not surprising as the association between recent morbidity, especially diarrhea, and acute growth faltering is well-established [25].

Receipt of MAD was significantly associated with both linear and ponderal infant growth in our cohort. Our findings support the existing and growing literature relating IYCF practices with child growth outcomes [24, 26]. However, we did not observe an association between child growth outcomes and its traditional predictors, such as household SES and maternal literacy [27, 28]. The lack of association between child growth measures and SES may be explained by the fact that in Bangladesh, prevalence of stunting is high even among the richest households [21].

Even though receiving MAD was strongly predictive of higher LAZ, infant diet did not meet the WHO recommendations even among the most food secure households in our study [29]. The main foods making up infant diet in our cohort were grains, roots and tubers, with only 10% of infants receiving eggs and vitamin A rich fruits and vegetables [29].

Although maternal literacy is an important determinant of IYCF practices [8, 30], we have not observed the same in this setting. Yu et al [16], working with the same population did not find maternal literacy to be a significant predictor of EBF at 3 months. Maternal literacy was also not associated with timely CF initiation in this setting (Owais et al, unpublished data). As maternal literacy likely impacts child growth outcomes through improved child rearing practices, including CF practices, and this association does not exist in our population, it is not surprising that we did not detect a link between maternal literacy and child growth outcomes in our cohort.

A major strength of our study is the large sample size which increases the power of our analysis. Furthermore, our ability to make causal inferences is strengthened as this was a prospective cohort study. However, as with any other observational study, this one also has some limitations. Due to the design of the program evaluation, we did not have information on the birthweight of infants included in our study. As low birthweight increases the risk of stunting in the first two years of life, not being able to adjust for it is a limitation of our study. Secondly, IYCF practices, the main exposures in our study, were determined based on maternal report and not directly observed feeding practices. This is a potential source of exposure misclassification. Furthermore, age at CF initiation is based on maternal recall several months after solid/semi-solid/soft foods were introduced to the infant. This time lag may also be a potential source of misclassification for this exposure, as maternal recall may have been affected. However, for EBF and MAD, maternal recall of what the child ate/drank over the past 24 hours was used to determine infant diet at 3 and 9 months, respectively. For age at CF initiation, we cross-referenced the reported age of complementary feeding initiation at the 9-month follow-up with infant diet (based on maternal recall) reported at the 3-month follow-up. A discrepancy was present for only 3% of infants included in this study and adjustments to the age at complementary feeding initiation were made accordingly.

In conclusion, this study adds to the growing body of longitudinal evidence linking IYCF practices and subsequent linear and ponderal childhood growth. Ensuring infants receive a diverse, high quality diet from 6 months onwards may mitigate both acute and chronic growth faltering in the second year of life. Nutrition programs aimed at improving child growth outcomes should include interventions, such as targeted educational messages, that can improve the quality of infant diet between 6–12 months of age.

## Supporting Information

S1 File Dataset. (ZIP)
